# Diffusion tensor image segmentation of the cerebrum provides a single measure of cerebral small vessel disease severity related to cognitive change

**DOI:** 10.1016/j.nicl.2017.08.016

**Published:** 2017-08-15

**Authors:** Owen A. Williams, Eva A. Zeestraten, Philip Benjamin, Christian Lambert, Andrew J. Lawrence, Andrew D. Mackinnon, Robin G. Morris, Hugh S. Markus, Rebecca A. Charlton, Thomas R. Barrick

**Affiliations:** a Neuroscience Research Centre, Molecular and Clinical Sciences Research Institute, St George's University of London, London, UK; b Department of Radiology, Charing Cross Hospital Campus, Imperial College NHS Trust, London, UK; c Stroke Research Group, Clinical Neurosciences, University of Cambridge, Cambridge, UK; d Atkinson Morley Regional Neuroscience Centre, St George's NHS Healthcare Trust, London, UK; e Department of Psychology, King's College Institute of Psychiatry, Psychology, and Neuroscience, London, UK; f Department of Psychology, Goldsmiths University of London, London, UK

**Keywords:** SVD, cerebral small vessel disease, DSEG, diffusion tensor image segmentation algorithm, EF, executive functions, IPS, information processing speed, Diffusion tensor imaging, Cognitive decline, Cerebral small vessel disease, Biomarker, Diffusion segmentation

## Abstract

Cerebral small vessel disease (SVD) is the primary cause of vascular cognitive impairment and is associated with decline in executive function (EF) and information processing speed (IPS). Imaging biomarkers are needed that can monitor and identify individuals at risk of severe cognitive decline. Recently there has been interest in combining several magnetic resonance imaging (MRI) markers of SVD into a unitary score to describe disease severity. Here we apply a diffusion tensor image (DTI) segmentation technique (DSEG) to describe SVD related changes in a single unitary score across the whole cerebrum, to investigate its relationship with cognitive change over a three-year period.

98 patients (aged 43–89) with SVD underwent annual MRI scanning and cognitive testing for up to three years. DSEG provides a vector of 16 discrete segments describing brain microstructure of healthy and/or damaged tissue. By calculating the scalar product of each DSEG vector in reference to that of a healthy ageing control we generate an angular measure (DSEG *θ*) describing the patients' brain tissue microstructural similarity to a disease free model of a healthy ageing brain. Conventional MRI markers of SVD brain change were also assessed including white matter hyperintensities, cerebral atrophy, incident lacunes, cerebral-microbleeds, and white matter microstructural damage measured by DTI histogram parameters. The impact of brain change on cognition was explored using linear mixed-effects models. Post-hoc sample size analysis was used to assess the viability of DSEG *θ* as a tool for clinical trials.

Changes in brain structure described by DSEG *θ* were related to change in EF and IPS (*p* < 0.001) and remained significant in multivariate models including other MRI markers of SVD as well as age, gender and premorbid IQ. Of the conventional markers, presence of new lacunes was the only marker to remain a significant predictor of change in EF and IPS in the multivariate models (*p* = 0.002). Change in DSEG *θ* was also related to change in all other MRI markers (*p* < 0.017), suggesting it may be used as a surrogate marker of SVD damage across the cerebrum. Sample size estimates indicated that fewer patients would be required to detect treatment effects using DSEG *θ* compared to conventional MRI and DTI markers of SVD severity.

DSEG *θ* is a powerful tool for characterising subtle brain change in SVD that has a negative impact on cognition and remains a significant predictor of cognitive change when other MRI markers of brain change are accounted for. DSEG provides an automatic segmentation of the whole cerebrum that is sensitive to a range of SVD related structural changes and successfully predicts cognitive change. Power analysis shows DSEG *θ* has potential as a monitoring tool in clinical trials. As such it may provide a marker of SVD severity from a single imaging modality (i.e. DTIs).

## Highlights

• A diffusion tensor image segmentation pipeline (DSEG) is proposed.
• The DSEG summary metric describes small vessel disease burden in a single score.
• DSEG is highly related to conventional markers of SVD (WMH, lacunes, CMB, atrophy).
• Change in DSEG over 3-years is highly related to change in executive function and processing speed.
• DSEG and lacunes are powerful markers for predicting SVD-related cognitive change.

## 1 Introduction

Cerebral small vessel disease (SVD) is a disease of the small perforating arteries and capillaries and results in tissue damage to the subcortical grey matter (GM) and white matter (WM) (Pantoni, 2010). SVD presents clinically with lacunar strokes (a result of blockage of the perforating arteries) and is the primary cause of vascular cognitive impairment (Wardlaw et al., 2013b). The course of SVD is heterogeneous and individuals may remain stable for a period of time or demonstrate a rapid decline in cognitive function (Lawrence et al., 2015). A goal of MRI research in SVD is to develop imaging biomarkers that can predict which individuals are at risk for greater decline, in order to target interventions at those who will benefit most.

MRI methods have been applied to measure brain changes and predict decline in function in SVD (Wardlaw et al., 2013a, Wardlaw et al., 2013b). T1-weighted images are typically used to examine whole brain atrophy as well as atrophy of grey matter (GM); T2-weighted or fluid-attenuated inversion recovery (FLAIR) images are used to quantify white matter hyperintensity (WMH) volume and presence of lacunar infarcts; T2* identify cerebral microbleeds (CMB); and diffusion tensor images (DTI) quantify white matter (WM) microstructure. Each of these measures has been associated with cognition in SVD, and particularly in the domains of executive function (EF) and information processing speed (IPS), which are affected early in the disease.

Whole brain atrophy is a feature of SVD, with the rate of volume loss exceeding that observed in healthy ageing (Nitkunan et al., 2011). Higher rates of atrophy are associated with increased decline in EF and IPS over a 3-year period (Jokinen et al., 2012). WMH and lacunar infarcts have also been associated with cognitive difficulties in SVD (Benisty et al., 2009, Benjamin et al., 2015, van den Heuvel et al., 2006, Lawrence et al., 2013, Schmidt et al., 2012). CMB have demonstrated a weak association with EF in SVD (Patel et al., 2013, Yamashiro et al., 2014). Decreased fractional anisotropy (FA) and increased mean diffusivity (MD) in normal appearing white matter is associated with poorer performance in EF and IPS, and associations with cognition are stronger for DTI metrics than for WMH, lacune volume and brain volume (Baykara et al., 2016, Nitkunan et al., 2008, O'Sullivan et al., 2004 & Tuladhar et al., 2015).

The relationships reported between single imaging biomarkers of SVD and cognition are often weak and research has suggested that combining multiple MRI metrics may provide the most accurate predictions of cognitive decline. For example, Baune et al. (2009) have shown that the presence of both WMH and incident lacunes has a larger negative impact on IPS and episodic memory (EM) than the presence of just one type of lesion. Supporting this, Jokinen et al. (2011) reported independent contributions of WMH and incident lacunes in predicting decline in EF and IPS. As MRI markers of SVD are often co-occur in individuals, recent efforts have been made to combine them into a unitary score of SVD burden (Huijts et al., 2013, Klarenbeek et al., 2013, Staals et al., 2015, Staals et al., 2014). Huijts et al. (2013) and Staals et al. (2014) used a score of SVD burden in which the presence of WMH, CMB, perivascular spaces and lacunar infarcts was summed to create a score between 0 and 4. Huijts et al. found that increased SVD burden as measured by this accumulation score was related to EF, IPS, EM and global cognition in participants at risk of SVD. Staals et al. (2015) used the same SVD burden score in a cohort of older participants and found that increases in combined MRI features of SVD were related to poorer general cognitive performance. They extended these findings by performing latent variable modelling to show that the four MRI features formed a unitary SVD construct and this latent construct was also related to cognitive performance.

However, multimodal MRI metrics require both longer scanning time (which may be uncomfortable for patients with SVD especially as cognitive difficulties increase) and complex post-processing (Wardlaw et al., 2013a, Wardlaw et al., 2013b). For a biomarker to be effective it should provide good predictive validity, be easy to acquire, and be useful across different scanners or sites (for use in multi-centre trials). Here, we propose a framework for the application of a whole cerebrum diffusion tensor image segmentation technique (Jones et al., 2014) that focuses on one imaging modality and, simultaneously includes isotropic and anisotropic diffusion metrics. The framework: i) provides information on whole brain microstructure that usefully describes a summary score related to brain tissue changes in SVD, ii) produces metrics that are associated with cognitive difficulties in SVD, and iii) provides useful predictive information regarding cognitive decline. In this study we apply a diffusion tensor image segmentation technique (DSEG) to a longitudinal sample of patients with SVD to examine differences in brain tissue diffusion profiles across the whole cerebrum, and associations with cognition. We hypothesise that across the cerebrum the percentage of DSEG segments representing healthy tissue will decrease over time, while segments representing damaged tissue will increase. We hypothesise that these changes will be related to change in EF and IPS. Finally we postulate that subject specific levels of GM atrophy and WM microstructural decline will be reflected in a summary metric, which will be significantly associated with decline in EF and IPS.

## 2 Methods

### 2.1 Participants

#### 2.1.1 SVD participants

Patients presenting with symptomatic SVD were recruited as part of the St George's Cognition And Neuroimaging in Stroke (SCANS) study. Recruitment occurred between 2007 and 2010 at three stroke services in hospitals covering a geographically contiguous area in South London, UK; St George's Hospital, King's College Hospital and St Thomas' Hospital. This data set has been described previously (Benjamin et al., 2015, Lambert et al., 2015, Lawrence et al., 2013, Zeestraten et al., 2016). Inclusion criteria comprised of a clinical lacunar stroke syndrome (Bamford et al., 1987) with radiological evidence of an anatomically corresponding lacunar infarct <= 1.5 cm diameter. In addition, inclusion criteria required confluent regions of WMH as graded two or more on the modified Fazekas scale (Fazekas et al., 1987, Hassan et al., 2003) and fluency in English sufficient to enable cognitive testing. Exclusion criteria were: contra-indications to undergo MRI scanning, any cause of stroke other than SVD (e.g. large artery stroke and cardioembolic stroke), current or history of central nervous system or major psychiatric disorder excluding migraine and depression, and any cause of white matter disease other than SVD. When new clinical strokes occurred during follow-up, patients remained eligible for the study providing the stroke was lacunar. Patients were recruited at least three months after last stroke occurrence to avoid the influence of any acute ischaemic effects on cognitive performance and MRI measures.

Patients were followed up annually with repeat MRI and cognitive testing for three years. At each follow-up visit, repeat recording of cardiovascular risk factors and blood pressure measurements was performed. The study was approved by the Wandsworth (London) research ethics committee and all patients provided written informed consent. The study was registered with the UK clinical research network (http://public.ukcrn.org.uk/, study ID: 4577).

#### 2.1.2 Available SVD data

At baseline a total of 121 patients were recruited. Of these 103 attended more than one cognitive assessment. Eighteen patients only attended one assessment due to death (n = 7), formal study withdrawal (n = 6), house move (n = 1), lost to follow-up (n = 2) and withdrawal from full neuropsychological testing (n = 2). Of the 103 patients who attended cognitive assessments more than once, MRI and neuropsychological data at multiple time points was available for 98 SVD patients (mean age = 68.42, SD = 9.98, range = 43–88, male = 65). Demographic baseline characteristics of the 98 patients who attended one or more follow-up are described in Table 1.

**Table 1:** Cerebral small vessel disease baseline risk factors and cognitive scores. A comparison between the longitudinal cohort and those that left the study after baseline assessment. Mean (standard deviation) or total (percentage) are shown.

	Longitudinal cohort (n = 98)	Baseline only (n = 23)	Test statistic
Age (years)	69.0 (9.93)	74.2 (7.76)	***t*(40.8) = − 2.7, *p* = 0.009**
Male sex	65 (66.3%)	13 (56.5%)	OR = 1.51, *p* = 0.500
Hypertension	91 (92.9%)	21 (91.3%)	OR = 0.81, *p* = 0.700
Statin therapy	84 (85.7%)	19 (82.6%)	OR = 0.79, *p* = 0.700
Diabetes	19 (19.4%)	5 (21.7%)	OR = 1.15, *p* = 0.800
Body mass index (kg/m^2^)	27.0 (5.19)	27.1 (3.08)	*t*(44.7) = − 0.1, *p* = 0.900
Current smoker	33 (33.7%)	9 (39.1%)	*p* = 0.800
Time since stroke (weeks)	26 (13.16)	104 (16.31)	*Z* = − 1.82, *p* = 0.069
Executive function z-scores	− 0.77 (1.00)	− 1.5 (0.91)	***t*(34.6) = 3.40, *p* = 0.002**
Information processing speed z-scores	− 0.74 (0.89)	− 1.1 (1.00)	*t*(28.5) = 1.60, *p* = 0.120

Significant results are shown in bold.

#### 2.1.3 Healthy ageing data

MRI data acquired was also available for a sample of healthy older adults. Participants were recruited to the St. George's Neuropsychology and Imaging in the Elderly (GENIE) longitudinal study. MRI data acquired at the third assessment was acquired using the same acquisition protocol on the same scanner as the SVD data. Full details of this sample have been described previously (Charlton et al., 2006, Charlton et al., 2013, Charlton et al., 2010). Briefly, 112 participants aged 50–90 years (mean age 69; 55 male) were recruited via local family doctors by random sampling, and were screened for MRI contraindications and prior psychiatric or neurological disorders; all spoke English as their first language. At the third time point (data included in the current DSEG analysis), good quality MRI data was available for 52 participants (mean age = 72.31, SD = 9.97, range = 53–91 years; male, n = 34) using the MRI acquisition as for the SVD data. The healthy ageing MRI data was included in the study to provide sufficient information for the DSEG technique to identify both healthy and damaged tissue in the DTI segmentation. Healthy ageing data was included to improve characterisation of the (*p*, *q*) space by providing a larger spectrum of diffusion profiles reflecting healthy to damaged brain tissue. The inclusion of healthy ageing controls in the DSEG processing also allowed for the use of a healthy ageing brain as the reference point in order to calculate DSEG *θ* (see Section 2.4.6). No group comparisons between healthy ageing and SVD patients were performed in this study. For group comparisons see Lawrence et al. (2013) where it was reported that there were no differences in age or gender between the SCANS and GENIE cohorts.

### 2.2 Magnetic resonance image acquisition

MR images were acquired using a 1.5-Tesla GE Signa HDxt system (General Electric, Milwaukee, WI, USA) with maximum gradient amplitude of 33 mT/m and a proprietary head coil. To standardise head position, patients were placed in a neutral position in the head coil with an alignment marker at the nasal bridge. To minimise head movement foam pads and a Velcro strap across the forehead were used. Total imaging time was approximately 45 min during which the following scan sequences, all providing whole head coverage, were obtained: 3D T1-weighted spoiled gradient recalled echo, FLAIR, T2*-weighted gradient recalled echo and single shot spin echo planar diffusion-weighted imaging (Benjamin et al., 2015, Lawrence et al., 2013, Zeestraten et al., 2016). Although the DSEG method described here relies on only DTI, other imaging modalities are included for comparison purposes.

DTI were acquired using the following parameters: acquisition matrix = 96 × 96, field of view (FOV) = 240 × 240 mm^2^, TE = 93.4 ms, TR = 15,600 ms, 55 slices without any slice gaps to provide an isotropic voxel resolution of 2.5 × 2.5 × 2.5 mm^3^, maximum b value = 1000 s mm^− 2^. Diffusion-weighted spin echo planar images were acquired with no diffusion weighting for eight acquisitions (i.e. b = 0 s mm^− 2^) followed by 25 non-collinear diffusion gradient directions and the negative of those diffusion gradient directions.

Axial FLAIR images: acquisition matrix = 256 × 192, FOV = 240 × 240 mm^2^, TI = 2200 ms, TE = 130 ms, TR = 9000 ms; slices = 28, slice thickness = 5 mm providing a voxel resolution of 0.45 × 0.45 × 5 mm^3^.

Spoiled gradient echo recalled T1-weighted (SPGR) 3D coronal sequence: image acquisition matrix = 256 × 192, FOV = 240 × 240 mm^2^, TE = 5 ms, TR = 11.5 ms, flip angle = 18°, slices = 176 coronal slices, slice thickness = 1.1 mm, providing isotropic voxel resolution of 1.1 mm^3^.

Axial T2*-weighted gradient echo: acquisition matrix = 256 × 192, FOV = 240 × 240 mm^2^, TE = 30 ms, TR = 300 ms, flip angle = 15°, 28 slices, slice thickness = 5 mm.

### 2.3 Magnetic resonance image analysis

#### 2.3.1 Diffusion-weighted image pre-processing

Following realignment of diffusion-weighted images to remove eddy current distortions using the FSL Linear Image Registration Tool (FLIRT, FMRIB Software Library, www.fmrib.ox.ac.uk/fsl) (Jenkinson and Smith, 2001), the acquired positive and negative diffusion gradient direction images (b = 1000 s/mm^2^) were geometrically averaged to eliminate gradient cross-terms (Neeman et al., 1991). The eight images without diffusion weighting (*b* = 0 s/mm^2^) were co-registered and averaged to give a T2-weighted echo planar image, henceforth referred to as the b_0_ image. Diffusion tensor maps were computed at each voxel using FSL DTIfit and isotropic *p* and anisotropic *q* maps were calculated for input to the DSEG algorithm (as described in Section 2.4.2).

The b0 images were skull stripped using FSL brain extraction software (BET: (Smith, 2002)) and the resulting brain mask applied to the *p* and *q* maps. The cerebellum was removed using an automated pipeline: i) a study specific template was generated by transforming the b_0_ image of a representative subject (defined as the participant with the median ventricular CSF volume) into the Montreal Neurological Institute standard space (i.e. MNI152 T_1_-weighted image) using a non-linear transform computed using Advanced Normalisation Tools (ANTS: (Avants et al., 2009)); ii) a mask covering the cerebellum and infratentorial brain stem was manually drawn over the study specific template using ITK-SNAP (Yushkevich et al., 2006); iii) each subject b_0_ image was non-linearly registered to the study specific template using ANTS and the inverse of this deformation field was used to warp the cerebellum mask into each individual native subject space; iv) any *p* and *q* voxels covered by the native space cerebellum masks were removed.

#### 2.3.2 Diffusion segmentation (DSEG) technique

Despite the prevalent use of DTI metrics, particularly FA as a marker of WM microstructure, the FA measurement does have limitations. Due to the way in which FA is calculated, proportional differences in the Euclidian magnitude of a tensor and the magnitude of its anisotropic component will result in the same FA value (Green et al., 2002) and consequently a given FA value will not be unique. This introduces a certain level of ambiguity in the interpretation of FA values for comparisons between participants, different tissue types or regions of interest. An alternative diffusion tensor decomposition represents isotropic (*p*) and anisotropic (*q*) components of the tensor as described by Pierpaoli and Basser (1996) and these are calculated as follows:

(1) $$p=\sqrt{3}\mathrm{MD},$$

(2) $$q=\sqrt{{\left(\lambda_{1}-\mathrm{MD}\right)}^{2}+{\left(\lambda_{2}-\mathrm{MD}\right)}^{2}+{\left(\lambda_{3}-\mathrm{MD}\right)}^{2}},$$

where

(3) $$\mathrm{MD}=\frac{\lambda_{1}+\lambda_{2}+\lambda_{3}}{3}.$$

Here, *p* is a scaled measure of the mean diffusivity and *q* is a measure of the deviation of the principal diffusivities from isotropy. These measures may be visualised in a 2D Cartesian plane, the (*p*, *q*) space, in which *p* and *q* have units in mm^2^ s^− 1^ (Peña et al., 2006) in a way that allows for differentiation of the diffusion properties of GM, WM tissue and cerebrospinal fluid (CSF).

Using this alternative (*p*, *q*) space representation of DTI data, Jones et al. (2014) developed and applied a novel DTI segmentation algorithm, DSEG. DSEG uses a *k*-medians cluster analysis to segment DTI data into 16 segments based on the magnitudes of the isotropic (*p*) and anisotropic (*q*) diffusion metrics for each voxel. Each segment describes a unique diffusion profile that represents the tissue microstructural properties of each voxel assigned to that segment. This allows differences in the underlying isotropic and anisotropic diffusion characteristics to be determined and compared between segments. The location of each segment can be used to identify brain regions with similar diffusion properties.

Once the segmentation is complete, the number of voxels assigned to each segment and/or the percentage of cerebral volume assigned to each segment can be computed, allowing the composition diffusion characteristics of the whole brain to be displayed as a spectrum for a given DTI dataset. This spectral information provides a signature diffusion profile containing information pertaining to GM and WM tissue, CSF, and also includes regions with diffusion profiles that deviate from those of healthy tissue. By considering the DSEG spectra as 16-dimensional vectors it is possible to calculate a summary metric as the scalar product, *θ*, between individuals. This summary metric can describe inter-subject differences in whole brain diffusion with respect to a reference brain, identified based on either behavioural or MRI measures.

In the present study, DSEG was performed simultaneously for all participants from the GENIE and SCANS studies using *p* and *q* maps as described by Jones et al. (2014). DSEG performs a k-medians clustering of the probability density function (i.e. 2D histogram) of *p* and *q* and is represented in (*p*, *q*) space. A *k*-medians algorithm was used (as opposed to k-means) as the 2D histogram of *p* and *q* values were non-Gaussian allowing the cluster centroids to be defined by the median.

As in Jones et al. (2014) *k* = 16 clusters are segmented. Each cluster provides a different characteristic diffusion property ranging from low anisotropy and isotropy (i.e. low *p* and *q* values mainly found in grey matter), high anisotropy and low isotropy (usually found in white matter) to high anisotropy and low isotropy (usually found in CSF). However, it is important to note that the DSEG is not an explicit tissue type segmentation and instead clusters voxels with similar diffusion properties. The choice of *k* = 16 clusters is pragmatic and is based on a optimising a balance between s separation of (*p*, *q*) space while avoiding over complication and statistical power issues by having too many segments. This is described in detail by (Jones, 2012).

**Fig. 1:**
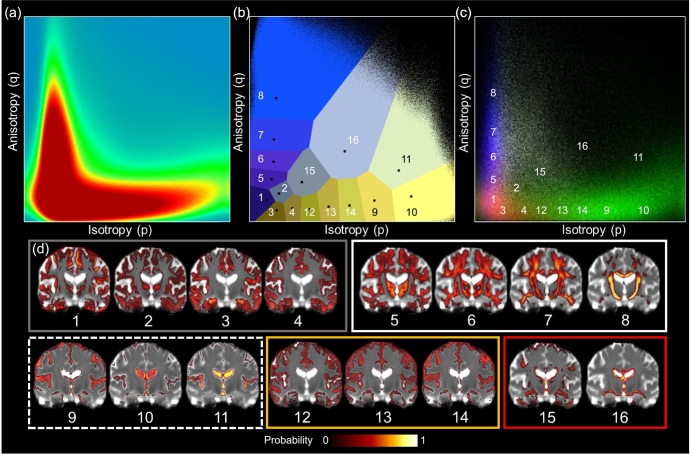
The result of DSEG segmentation of the whole brain in the SCANS SVD cohort. (a) Shows the 2D histogram of *p* and *q* profiles from the whole longitudinal cohort, including healthy ageing individuals. (b) Shows the segmented (*p*, *q*) space. (c) Represents the 2D-histogram of *p* and *q* values for different tissue classes (GM: red, WM: blue, CSF: green & WMH: white). (d) Shows the probability maps showing the location in standard space that each segment is most likely to be found in the cerebrum, grouped into GM (grey box), WM (white box), CSF (dashed white line box), GM/CSF boundary (yellow box) and damaged tissue segments (red box). (For interpretation of the references to colour in this figure legend, the reader is referred to the web version of this article.)
Step 2: *p* and *q* maps are considered as a set of observations (*X*_1_, *X*_2,_ … *X*_*n*_) where each observation is a 2D vector in (*p*, *q*) space. *K*-medians clustering partitions the observations into *k* disjoint subsets *S*_*j*_, where *j* = (1, 2, … *k*) by minimising the within-cluster sum of squares objective function,

The following steps describe the application of *k*-medians clustering to *p* and *q* maps:

Step 1: Histograms of *p* and *q* were computed using all brain voxels from the cross-sectional GENIE and longitudinal SCANS data. High and low intensity noise was removed from each distribution by computing the 1st and the 99.99th percentiles and assigning values below and above these thresholds to zero or one, respectively. Voxels surviving this threshold were then scaled between zero and one, resulting in non-Gaussian *p* and *q* histograms of the whole data set (Fig. 1a).

(4) $$J=\sum_{j=1}^{K}\sum_{n\in S_{j}}{\left\|X_{n}-\mu_{j}\right\|}^{2},$$

where *X*_*n*_ is a vector representing the nth data point and *μ*_*j*_ is the geometric centroid (i.e. median) of the data points in *S*_*j*_. The initial clusters were defined by separating the (*p*, *q*) space into *k* clusters of roughly equal size according to median and quartile values of *p* and *q*. These initial segments reflect the non-Gaussian structure present in the *p* and *q* histograms (Fig. 1a).

The following two steps are repeated to iteratively assign all voxels to one of *k* clusters and then recalculate cluster centroids.

Step 3: Each voxel was assigned to the nearest cluster centroid in (*p*, *q*) space. As a result voxels are partitioned into *k* clusters given below at the tth iteration,

(5) $$S_{i}^{\left(t\right)}=\left\{X_{j}:\left\|X_{j}-\mu_{i}^{\left(t\right)}\right\|\leq \left\|X_{j}-\mu_{i^{*}}^{\left(t\right)}\right\|\;\;\mathrm{for}\;\mathrm{all}\;i^{*},i\in \left\{1,\ldots ,k\right\}\right\}.$$

Step 4: Median *p* and *q* values are recalculated.

The DSEG algorithm is terminated when there is no longer any movement of voxels between clusters or at 250 iterations, at which point voxel classification of diffusion properties across the different segments is stable. The resulting segmentation of (*p*, *q*) space is represented in the Voronoi plot shown in Fig. 1b. Fig. 1c shows the (*p*, *q*) histogram distributions for voxels separated by the following tissue classes as segmented using methods described by Lambert et al. (2015); GM (red), WM (blue), WMH (white) and CSF (green). Comparison of Fig. 1b and c show how the DSEG segments map onto these basic tissue types.

#### 2.3.3 DSEG probability maps

DSEG maps were transformed into the study specific template space and the mean spatial placement of each segment was calculated over the whole cerebrum to provide maps showing the likelihood of diffusion profiles being located in each brain voxel (i.e. a spatial probability map). These probability maps are shown in Fig. 1d and add support to the interpretation of DSEG segments having diffusion profiles that represent different tissue types.

#### 2.3.4 DSEG colour maps

**Fig. 2:**
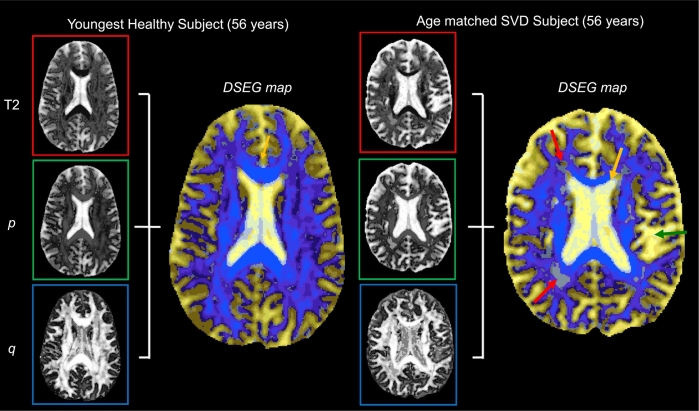
Two examples of how DSEG colour maps are generated using the scaled rank scores of signal from T2-weighted, p and q images. (a) Represents the DSEG map of the youngest healthy subject (56 years) used as the reference brain in DSEG *θ* calculations. (b) Represents age-matched subject with SVD (56 years). The orange arrow shows how the corpus callosum is represented by segment 8 while the red arrows highlight areas identified as WMH on FLAIR images represented by segments 15 and 16. The green arrow indicates greater volume of CSF space in the sulci in the SVD patient compared to the control. (For interpretation of the references to colour in this figure legend, the reader is referred to the web version of this article.)

DSEG maps were visualised using a unique RGB colour scheme to display the relative magnitude of *p* and *q* metrics and T2-weighting (obtained from the b_0_ maps) in each segment (Jones et al., 2014). The median values for *p*, *q* and T2-weighting within each segment were ranked from 1 (lowest) to 16 (highest) and the rank scores were used to generate RGB colours by assigning T2-weighting to the red channel, *p* to the green channel and *q* to the blue channel. Colour maps were visualised using MRIcro (Rorden and Brett, 2000). Fig. 2 illustrates how isotropic and anisotropic diffusion measures are combined with information from T2-weighted images and visualised for the youngest healthy subject (Fig. 2a: age 56 years from the GENIE study) and an age matched SVD patient (Fig. 2b: age 56 years). DSEG colour maps provide visual information about the diffusion properties within the tissue microstructure at each voxel. Orange arrows in Fig. 2 indicate the highly organised corpus callosum, which is characterised by high anisotropy and low isotropy as represented by segment 8 on the Voronoi plot (Fig. 1b) and the highlighted area for the segment 8 probability map (Fig. 1d). Red arrows indicate WMH regions (identified on FLAIR) on the DSEG colour map (Fig. 2b). These WMH regions are characterised by grey colours (segments 15 and 16 on the Voronoi plot, Fig. 1b) and represent white matter regions with lower anisotropy and higher isotropy than healthy white matter tissue.

#### 2.3.5 DSEG whole brain spectra

To generate individual DSEG spectra for each participant, the number of cerebrum voxels within each DSEG segment was determined and the percentage contribution of each segment to the total cerebrum volume was calculated. This provides a subject specific diffusion profile referred to as a DSEG spectrum.

#### 2.3.6 DSEG summary metric

The angle, *θ*, between two vectors **A** = (*a*_1_, *a*_2_, …, *a*_16_) and **B** = (*b*_1_, *b*_2_, …, *b*_16_) may be given by the scalar product,

(6) $$\theta =\cos^{-1}\left(\frac{A∙B}{\left\|A\right\|\left\|B\right\|}\right),$$

Eq. (6) provides a summary metric for the difference between two DSEG spectra that are represented by vectors **A** and **B**.

To ensure the metrics may be compared across subjects, vector **A**, was chosen to represent the DSEG spectrum representing the ‘least damaged’ brain. This reference brain was identified using an iterative algorithm. Initially, *θ* was calculated between a randomly selected DSEG spectrum (labelled vector **A**) and each remaining spectrum in the dataset (labelled vector **B**). The spectrum with maximum *θ* was identified and relabelled as vector **A**. The process was repeated until vector **A** oscillated between two DSEG spectra (i.e. the spectra of the “least” and “most damaged” individuals). We identified vector **A** to represent the “least damaged” individual. This corresponded to the DSEG spectrum of the youngest participant in the GENIE sample (aged 56 years). Vector **B** was then used to represent the DSEG spectra for each individual at each time point in order to calculate *θ* for all individuals at each time point. The scalar product method is similar to the functional correlation measures used in several resting state functional MRI studies (Van Dijk et al., 2010).

### 2.4 Conventional image analysis

To allow comparison of the performance of the DSEG *θ* measure to conventional imaging biomarkers in SVD several measures were determined from the T1-weighted, FLAIR and T2*-weighted images as well as conventional DTI histogram measures. Measures included total cerebral volume (TCV), white matter hyperintensity lesion load (WMH load), number of lacunes and number of cerebral microbleeds (CMB).

#### 2.4.1 Structural pre-processing

A longitudinal tissue segmentation pipeline optimised to our symptomatic SVD cohort was performed to obtain tissue probability maps for GM, normal appearing white matter (NAWM), WMH and CSF from FLAIR and T1-weighted images. This is described in detail in Lambert et al. (2015) and Zeestraten et al. (2016) and provides total cerebral volume, WMH load and number of lacunes.

#### 2.4.2 Total cerebral volume

Volumes of GM, NAWM and WMH were calculated in the native space from a hard segmentation of the tissue probability maps. For each participant and time point TCV in millilitres was calculated as the sum of GM, NAWM and WMH volumes.

#### 2.4.3 WMH load

WMH load for each patient and time point was calculated as the percentage of WMH volume within the TCV. WMH volume was calculated from the hard segmentation of the tissue probability maps. The performance of the final semi-automated binary thresholding of the WMH tissue probability maps segmentation maps provided an intra-class correlation coefficient of 0.99 across a subset of 20 randomly selected patients by two raters, EAZ and CL.

#### 2.4.4 Lacunes

A consultant neuroradiologist (ADM) evaluated T1-weighted and FLAIR scans for lacunes. To ensure an underlying SVD aetiology of lacunes and exclusion of perivascular spaces the lacunes were defined as CSF filled cavities of 3–15 mm in diameter with a surrounding rim of FLAIR hyperintensity (Wardlaw et al., 2013a, Wardlaw et al., 2013b). Follow-up scans for each patient were registered to a group average template, as described in Lambert et al. (2015), allowing accurate identification of incident lacunes. Lacune numbers at each time point were determined. Lacune reliability metrics were checked across a subset of 20 randomly selected patients assessed by the consultant neuroradiologist (ADM) and an additional rater (EAZ) and provided an intra-class correlation coefficient of 0.99.

#### 2.4.5 Cerebral microbleeds

CMB were defined as homogeneous round focal areas < 10 mm in diameter of low signal intensity on T2*-weighted GRE images. Only CMB meeting the Brain Observer Microbleed Rating Scale (BOMBS) (Cordonnier et al., 2009) criteria for “certain” CMB were analysed. Presence and number of new CMB were noted for all patients. All baseline CMB were identified by a single consultant neuroradiologist (ADM). CMB on follow-up were identified by a single rater (EAZ). Using scans of a subset of 10 randomly selected patients that were assessed by both raters the intra-class correlation coefficient was 0.99.

#### 2.4.6 Conventional DTI histogram measures

Conventional DTI measure histograms were also computed for whole brain WM (including both NAWM and WMH) as described in Zeestraten et al. (2016). These included histogram measures calculated for the mean diffusivity (MD) and fractional anisotropy (FA) metrics. Normalised histograms were computed in a composite mask containing NAWM and WMH. The WM tissue masks (in the native T1-weighted image space) were co-registered to the *b*0 image using FMRIB Non-linear Image Registration Tool (FNIRT) (Andersson et al., 2007). Based on a diffusivity threshold, spurious CSF voxels within the WM masks were removed (i.e. voxels with MD > 0.0026 mm^2^ s^− 1^. Histograms of MD (range 0–0.004 mm^2^ s^− 1^, bin width 0.000004 mm^2^ s^− 1^) and FA (range 0–1, bin-width 0.001) histograms were computed and median and normalised peak frequency (i.e. normalised peak height, NPH). The NPH of MD has previously been identified as the most stable and sensitive DTI histogram measure to change of tissue microstructure in SVD (Zeestraten et al., 2016).

### 2.5 Cognitive assessment

A battery of standardised neuropsychological tasks sensitive to the cognitive impairments seen in SVD was carried out annually. Details of the full assessment have been published previously (Lawrence et al., 2013, Lawrence et al., 2015) but this study will only consider the domains shown to be most severely affected in SVD, namely EF and IPS (Lawrence et al., 2013, Pantoni, 2010, Zhou and Jia, 2009) EF was measured by the Trail Making Test, part B (Reitan, 1958), Letter Fluency (Delis et al., 2001), and the modified Wisconsin Card Sorting Test (categories completed and perseverative errors) (Nagahama et al., 2003). IPS was measured by Digit Symbol Substitution (Wechsler, 1997), the Grooved Pegboard Test (Klove, 1963, Mitrushina et al., 2005), and the BIRT Memory and Information Processing Battery (BMIPB) Speed of Information Processing test: Participants were presented with a series of five two-digit numbers and asked to identify the second to highest number in each series. The measure derived from this test is the total of correctly completed series within a four minute time limit, corrected for by the time it takes to complete a matching motor task (Coughlan et al., 2007). Individual measures were age-scaled using published normative data, converted to z-scores and a mean composite cognitive domain score was calculated within each domain (EF and IPS). Parallel test forms were employed for two tests to reduce learning effects: the B-MIPB speed of information processing task (4 forms) and single letter verbal fluency (annually alternating F-A-S and B-H-R). All other tasks were identical at each assessment. Premorbid intelligence (IQ) was assessed using the National Adult Reading Test-restandardised (NART) (Nelson and Willison, 1991) and the Mini Mental State Exam (MMSE) (Folstein et al., 1975) was used as a dementia screening tool.

### 2.6 Statistical analysis

Baseline differences between patients who remained in the study and those who left the study have been described before in Lawrence et al. (2015). Analysis is replicated here. Welch's *t*-tests was used for continuous variables, Fisher's exact test for categorical data (i.e. smoking status) and odds-ratios for 2 × 2 categorical data.

Linear mixed-effects (LME) models were applied using MLwiN (Rasbash et al., 2009) and used to assess the effects of time on change in cognition, DSEG, DTI and structural markers. LME models were used due to the hierarchical nature of the data, with each parameter having multiple measurements per subject. The trajectories of each measure for each individual were modelled as a linear trend across the follow-up period as a function of time since baseline cognitive testing or scanning. The intercept and slope of each participant's linear trajectory were allowed to vary with both fixed and random effects. Fixed effect variation was accounted for by time, and random effect variation allowed for remaining inter-individual differences. The average fixed effects slopes of time represent the average annualised change rate for a given measure. The change rates for each measure (cognitive or imaging) were evaluated using the Wald test, which assesses the goodness of fit between the observed values and the expected values (i.e. the modelled slope). The Wald test takes on a *χ*^2^ distribution, which is used to calculate the statistical significance of each model.

LME models were also used to assess the relationships between change in individual DSEG segmented and the whole cerebrum DSEG *θ*. Further LME models were used to assess the relationships between the effects of change in imaging measures on cognitive domains, with fixed effect variation being accounted for by change in MRI measures with time. The average fixed effects slopes of MRI measures represent the average annualised change rate for a given cognitive domain related to one unit change in the MRI measure. Univariable models assessed the impact of MRI markers on cognitive domains in the first instance and those markers that were significantly related to change in cognition were included in a set of multivariable analyses. This was to ensure that only markers that are independently related to cognitive change were included in multivariable models. Covariates including age, gender and time since stroke were also only included if there were significantly related to EF or IPS in univariable analysis. For the secondary, multivariable analysis, a more conservative significance level was set using Bonferroni correction for multiple comparisons.

Post-hoc sample size estimations were performed for DSEG *θ* to assess its potential use in a clinical trial setting. Sample size estimates for the number of patients required at each time point in a 3-year annual testing trial was calculated using the longpower statistical package in R (version 3.02: www.R-project.org) (Liu and Liang, 1997). The minimum sample sizes that were required to detect a change in the rate of DSEG *θ* were estimated for effect sizes of 30%, 25%, 20% and 15%. A balanced trial was assumed with a power of 80% and α = 0.05.

## 3 Results

### 3.1 Baseline comparison between longitudinal cohort and those who did not return for follow-up

Table 1 shows the baseline characteristics of the longitudinal cohort (n = 98) compared to all patients who did not attend follow-up testing (n = 23). The longitudinal cohort was significantly younger (mean age = 69.00, S.D. = 9.93) than the dropout group (mean age = 74.20, S.D. = 7.76). The longitudinal cohort also performed significantly better in EF tasks (mean EF = − 0.77, S.D. = 1.0) compared to the dropout group (mean EF = − 1.50, S.D. = 0.91). There were no other significant differences in baseline characteristics between the longitudinal cohort and the dropout group.

### 3.2 Brain tissue characterisation using DSEG maps

The final segmentation of (*p*, *q*) space can be seen in Fig. 1b and *p* and *q* median values for each DSEG segment are given in Supplementary Table 1. Fig. 1b and the accompanying probability maps in Fig. 1d suggest that segments 1–4 represent GM and have characteristic diffusion signatures with low *p* (isotropy) and low *q* (anisotropy). WM is represented by segments 5–8, and have diffusion signatures with low *p* and increasing *q*, identifying increasingly organised microstructure. For example, more densely packed axons or uniform axonal orientation are represented by segment 8 in the corpus callosum and the corticospinal tracts. The CSF is represented by segments 9–11 with high isotropy diffusion signatures. Segments 12–16 include voxels at tissue boundaries: segments 12–14 are at the boundary of cortex and CSF space and segments 15–16 are located at the border between WM and the lateral ventricles. These segments are likely to include tissue partial volume effects that may suggest GM (segments 1–4) and WM (segment 5–7) atrophy. We define segments 12–16 as intermediate segments as they represent diffusion characteristics that deviate from healthy tissue signatures (i.e. with higher *p* and lower *q* than healthy white matter segments) and may indicate damaged or abnormal tissue.

### 3.3 DSEG spectra

**Fig. 3:**
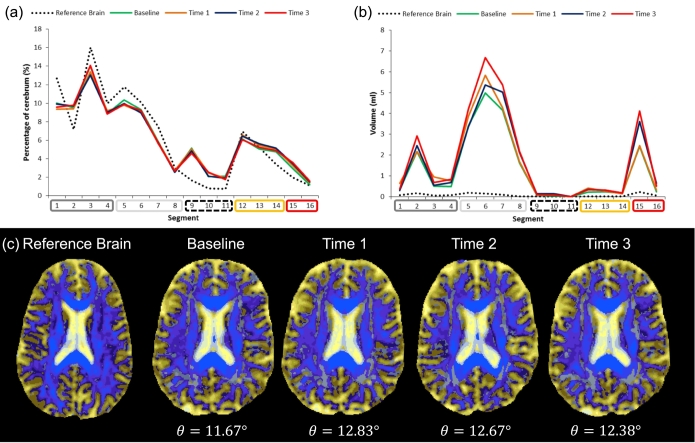
An example of an individual who did not show steep increases in DSEG *θ*. At baseline this patient was 61 years old, 2.25 years since time of stroke, had an EF z-score of − 1.79 and an IPS z-score of − 0.90. (a) Represents the DSEG spectra of the whole cerebrum at each time point and can be compared to the spectrum of the reference brain. The spectra show minimal change, suggesting brain microstructure stability in this individual over time. (b) The spectra extracted from WMH show minimal change over time. In these graphs, the Reference brain spectrum is indicated by the black dotted line, Baseline by the green line, Time 1 by the orange line, Time 2 by the blue line and Time 3 by the red line. (c) Example axial DSEG slices are shown using the radiological convention at each time point with the corresponding DSEG *θ* value. As suggested by the spectra, changes in the DSEG images are minimal. (For interpretation of the references to colour in this figure legend, the reader is referred to the web version of this article.)

**Fig. 4:**
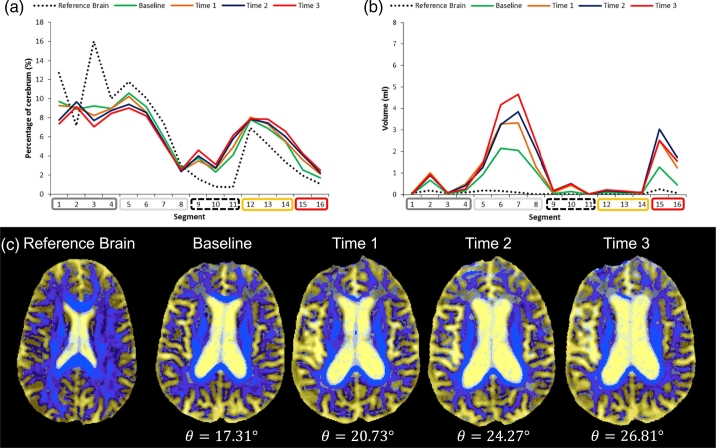
An example of an individual who exhibited a steep increase in DSEG *θ* over time. At baseline this patient was 65 years old, 2.31 years since time of stroke, had an EF z-score of − 1.03 and an IPS z-score of − 0.14. (a) Represents the DSEG spectra at each time point compared to the reference brain spectra. The spectra change each year, becoming more different to the reference. (b) The spectra extracted from WMH show change over time indicating an increase in the volume of WMH. In these graphs, the Reference brain spectrum is indicated by the black dotted line, Baseline by the green line, Time 1 by the orange line, Time 2 by the blue line and Time 3 by the red line. (c) Example axial DSEG slices are shown using the radiological convention at each time point with the corresponding DSEG *θ* value. As suggested by the spectra, changes in the DSEG images are substantial. (For interpretation of the references to colour in this figure legend, the reader is referred to the web version of this article.)

Fig. 3, Fig. 4 illustrate DSEG spectra for the reference brain (dotted line) and an example of one individual who changed little (Fig. 3) or changed substantially (Fig. 4) over the three year study period. As can be seen in Fig. 3a, the spectra profile of the SVD patients deviates from the healthy ageing reference spectra particularly in segments 1–4 (relating to GM) and segments 5–6 (relating to WM). However spectra of the segments do not demonstrate a substantial change over the 4 available time points, as can be seen in the stability of DSEG *θ* (Fig. 3c). Fig. 3b illustrates the spectra within WMH which is substantially different between the reference brain and the SVD patient. The differences in WMH spectra are particularly pronounced in segments 5–8 (relating to WM), but also in segment 2 (relating to deep GM) and segment 15 (at the boundary of lateral ventricles). Fig. 4 illustrates an individual who changed substantially over the study period. Not only does the spectra of this individual differ from the reference spectra (Fig. 4a) in most segments with the exception of segment 8 (representing highly organised corticospinal tracts), spectra also changed from baseline to Time 3. This difference is also observed in the summary metric DSEG *θ* (Fig. 4c). Spectra within WMH differ substantially from the reference spectra particularly in segments 5–8 (representing WM) and segments 15–16 (boundary segments, see Fig. 4b), and also demonstrated substantial increase longitudinally. Table 2 shows the relationship between change in DSEG segments as a percentage of total cerebrum volume and the summary metric, DSEG *θ.* It can be seen that an increase in DSEG *θ* is associated with a decrease in healthy GM (segments 1–4) and WM (segments 5–7) and an increase in CSF space (segments 9–11), partial volume tissue at the border of GM and CSF (segments 12–14), and an increase in WMH related damage (segments 15 &16). As such DSEG *θ* successfully describes changes in microstructure over a three-year period in one measure.

**Table 2:** LME results showing the relationships between annual change in DSEG segments and change in DSEG *θ*. Beta values represent the average change in DSEG *θ* given a percentage unit change for each segment. S.E. = standard error.

Segment	Beta	S.E.	*χ*^2^, *p*
1 (GM)	**− 1.173**	**0.097**	**146.533, < 0.001**
2 (GM)	**1.278**	**0.220**	**33.861, < 0.001**
3 (GM)	**− 2.357**	**0.090**	**685.551, < 0.001**
4 (GM)	**− 0.388**	**0.270**	**2.058, 0.151, < 0.001**
5 (WM)	**− 3.505**	**0.253**	**192.017, < 0.001**
6 (WM)	**− 3.163**	**0.385**	**67.163, < 0.001**
7 (WM)	**− 1.874**	**0.443**	**17.941, < 0.001**
8 (WM)	− 0.435	0.637	0.466, 0.495
9 (CSF)	**2.411**	**0.173**	**194.149, < 0.001**
10 (CSF)	**2.412**	**0.187**	**165.851, < 0.001**
11 (CSF)	**1.255**	**0.255**	**24.202, < 0.001**
12 (GM/CSF border)	**0.872**	**0.361**	**5.840, 0.016**
13 (GM/CSF border)	**3.351**	**0.300**	**124.895, < 0.001**
14 (GM/CSF border)	**3.149**	**0.245**	**164.563, < 0.001**
15 (Damaged tissue)	**2.028**	**0.171**	**140.636, < 0.001**
16 (Damaged tissue)	**4.097**	**0.547**	**56.182, < 0.001**

Significant results shown in bold, after Holm-Bonferroni correction.

### 3.4 Linear mixed effect models of cognitive and brain structural decline over three years

Table 3 shows the fixed effects results of LME models of change in cognition and imaging metrics over time. As time was measured in years, the beta values indicate the average annual change for each variable. Using this conservative estimation, neither EF nor IPS showed significant decline over a three-year period. In contrast, all MRI markers demonstrated a significant decline over three years. DSEG *θ* declined significantly, suggesting a decrease in whole brain microstructural health causing the difference between individual DSEG spectra and that of the reference brain becomes smaller. Decreases in WM microstructural integrity are apparent with significant decreases in MD NPH and FA median and an increase in MD median. FA NPH did not change significantly. WMH load increased over time while TCV decreased with time, indicating increased levels of atrophy. In total 74 new lacunes were observed in 27 patients, with a single new lacune being found in 10 patients, two new lacunes in nine patients and three or more in eight patients. There were 173 new CMBS found in 35 patients with a single new CMB found in 10 patients, 2–5 new CMBs found in 14 patients and six or more CMBs found in 11 patients. New lacunes and CMB were log transformed due to non-normal distributions in order to allow the inclusion of these metrics in LME models.

**Table 3:** Linear mixed effect models of change in cognition and magnetic resonance imaging biomarkers over a period of three years. Beta values represent the average annual rate of change for each variable, S.E. = standard error.

	Beta	S.E.	*χ*^2^, *p*
Cognitive variables			
EF	− 0.016	0.021	0.582, 0.445
IPS	− 0.005	0.019	0.079, 0.779
DSEG			
θ	**1.168**	**0.085**	**190.149, < 0.001**

DTI histogram measures			
MD NPH	− 3.17 × 10^− 4^	3.11e × 10^− 7^	142.355, < 0.001
MD median (mm^2^ s^− 1)^	5.29 × 10^− 6^	5.09 × 10^− 7^	108.047, < 0.001
FA NPH	6.84 × 10^− 7^	5.08 × 10^− 6^	0.018, 0.893
FA median	−** 0.002**	**0.0004**	**28.802, < 0.001**

Conventional MRI measures			
WMH load	**0.773**	**0.064**	**145.748, < 0.001**
TCV (ml)	−** 13**,**830.838**	**810.366**	**291.296, < 0.001**
Lacunes	**0.019**	**0.004**	**17.703, < 0.001**
CMB	**0.034**	**0.005**	**38.004, < 0.001**

Significant results shown in bold.

Table 4 shows the relationship between change in DSEG *θ* and change in conventional markers of SVD and DTI histogram measures related damage in LME models. DSEG *θ* was significantly associated with all conventional markers and DTI histogram measures suggesting that as DSEG *θ* increases there are higher rates of atrophy, WMH load, incident lacunes, CMB and a decline in WM microstructure.

**Table 4:** Univariable linear mixed effect models of change in DSEG *θ* related to change in conventional magnetic resonance imaging biomarkers of SVD and DTI histogram measures over a period of three years.

	Beta	S.E.	*χ*^2^, *p*
DTI histogram measures			
MD NPH	− 1169.333	140.115	**69.648, < 0.001**
MD median (mm^2^ s^− 1^)	81,309.227	8070.304	**101.508, < 0.001**
FA NPH	4813.334	857.420	**31.514, < 0.001**
FA median (mm^2^ s^− 1^)	− 28.673	11.997	**5.712, 0.017**

Conventional MRI markers			
WMH load	0.479	0.136	**12.341, < 0.001**
TCV (ml)	1.48 × 10^− 5^	5.40 × 10^− 6^	**7.492, 0.006**
Lacunes	5.015	1.255	**15.967, < 0.001**
CMB	2.636	1.037	**6.470, 0.011**

Significant results shown in bold.

S.E. = Standard error.

### 3.5 Univariable linear mixed effect models of the impact of change in brain structure on change in cognition

Initial assessment of the impact of brain change on decline in cognition was carried out using univariable LME models. Table 5, Table 6 show the results for EF and IPS in which the beta values are related to the predicted change in a given cognitive domain for every unit change in the MRI marker. For example, one degree increase in DSEG *θ* is predicted to be associated with a change of − 0.047 z-scores in EF. Thus increases in DSEG *θ* will have a negative impact on EF performance over time. Change in EF was also related to change in MD NPH, MD median, FA median, TCV, and new lacunes. IPS was related to DSEG *θ*, MD NPH, MD Median, FA NPH, FA median, new lacunes and CMBs. Both cognitive domains were related to premorbid IQ; IPS was also related to sex, but neither domains were related to age or years since stroke.

**Table 5:** Univariable and multivariate linear mixed effect models of executive function change related to change in magnetic resonance imaging biomarkers.

EF	Univariable models		Multivariable MRI and IQ	
	Beta (S.E.)	*χ*^2^, *p*	Beta (S.E.)	*χ*^2^, *p*
DSEG *θ*	**− 0.047 (0.010)**	**23.205, < 0.001**	**− 0.036 (0.011)**	**11.208, < 0.001**
MD NPH	**79.6 (28.400)**	**7.860, 0.005**	23.288 (38.009)	0.375, 0.540
MD Med (mm^2^ s^− 1^)	**− 4.91 × 10**^**3**^**(1.86 × 10**^**3**^**)**	**6.980, 0.008**	3232.074 (2881.822)	1.258, 0.262
FA NPH	− 123.073 (227.579)	0.295, 0.0587	a	a
FA Med (mm^2^ s^− 1^)	**4.999 (2.551)**	**3.840, 0.050**	− 0.531 (3.973)	0.018, 0.894
WMH load	− 0.051 (0.027)	3.488, 0.062	a	a
TCV (ml)	**2.17 × 10**^**− 6**^**(8.84 × 10**^**− 7**^**)**	**6.060, 0.014**	1.01 × 10^− 6^ (6.74 × 10^− 7^)	2.236, 0.135
Lacunes	**− 0.606 (0.210)**	**8.302, 0.004**	**− 0.544 (0.175)**	**9.613, 0.002**
CMB	− 0.328 (0.172)	3.636, 0.057	a	a
Age	− 0.012 (0.010)	1.286, 0.257	a	a
IQ	**0.045 (0.005)**	**76.880, < 0.001**	**0.043 (0.005)**	**90.733, < 0.001**
Sex	− 0.284 (0.219)	1.681, 0.194	a	a
Years stroke	− 0.037 (0.021)	3.058, 0.080	a	a

Significant results shown in bold.

S.E. = standard error.

a Indicates variable was not included in multivariable model.

**Table 6:** Univariable and multivariate linear mixed effect models of information processing speed change related to change in magnetic resonance imaging biomarkers.

IPS	Univariable models		Multivariable MRI and IQ	
	Beta (S.E.)	*χ*^2^, *p*	Beta (S.E.)	*χ*^2^, *p*
DSEG *θ*	**− 0.029 (0.008)**	**14.5, < 0.001**	**− 0.031 (0.010)**	**10.648, 0.001**
MD NPH	**60.842 (22.958)**	**7.023, 0.008**	3.071 (34.759)	0.008, 0.930
MD Med (mm^2^ s^− 1^)	**− 3528.697 (1503.716)**	**5.510, 0.019**	7063.002 (2775.184)	6.477, 0.011
FA NPH	**− 591.526 (180.716)**	**10.714, < 0.001**	− 496.639 (212.073)	5.484, 0.019
FA Med (mm^2^ s^− 1^)	**4.796 (2.041)**	**5.523, 0.019**	5.869 (3.437)	2.916, 0.088
WMH load	− 0.033 (0.022)	2.311, 0.128	a	a
TCV (ml)	7.99 × 10^− 7^ (7.1 × 10^− 7^)	1.270, 0.260	a	a
Lacunes	**− 0.715 (0.160)**	**19.934, < 0.001**	**− 0.509 (0.165)**	**9.497, 0.002**
CMB	**− 0.275 (0.143)**	**3.719, 0.054**	0.028 (0.144)	0.037, 0.847
Age	0.015 (0.008)	3.665, 0.056	a	a
IQ	**0.021 (0.005)**	**17.778, < 0.001**	**0.018 (0.004)**	**19.578, < 0.001**
Sex	**− 0.482 (0.163)**	**8.733, 0.003**	− 0.337 (0.137)	6.087, 0.014
Years stroke	− 0.022 (0.025)	0.809, 0.368	a	a

Significant results shown in bold.

S.E. = standard error.

a Indicates variable was not included in multivariable model.

### 3.6 Multivariable linear mixed effect models of change in MRI biomarkers related to cognitive change

All MRI markers that were significantly (*p* = 0.05, uncorrected) related to a cognitive domain in univariable analysis were subsequently entered into a multivariable analysis in order to assess the relative contribution of each marker to cognitive change when all other markers are controlled for. A multivariable LME model was created for EF and IPS in turn, in which MRI markers and premorbid IQ, age and years since stroke were included if they were significantly related to each cognitive domain.

#### 3.6.1 EF

The multivariable model for EF is shown in Table 5. It can be seen that the only MRI markers to remain significant in the multivariable model after correction for multiple comparisons was DSEG *θ* and number of new lacunes. A degree change in DSEG *θ* predicted a − 0.036 z-score change in EF. New lacunes was related to a − 0.544 change in EF, though the direct impact of each new lacune is unclear due to log transformation of the data. Premorbid IQ was also related to change in EF, where a unit increase in premorbid IQ predicted slower rate of change in EF by 0.043 z-scores.

#### 3.6.2 IPS

The multivariable model for IPS (shown in Table 6), demonstrated that DSEG *θ*, and new lacunes were the only MRI markers significantly related to IPS after correction for multiple comparisons. A degree increase in DSEG *θ* predicted − 0.031 z-score change in IPS. A unit change in lacunes predicted a change of − 0.509 z-score in IPS. A unit difference in premorbid IQ related change in IPS, where a unit increase in premorbid IQ predicted slower rate of change in IPS by 0.019 z-scores.

### 3.7 Post-hoc sample size estimates for DSEG *θ*

Table 7 shows the results of the post-hoc sample size estimates for DSEG *θ* compared to the best performing conventional and diffusion imaging markers of SVD progression previously reported in the same SCANS data over 3-years (Benjamin et al., 2015 & Zeestraten et al., 2016). Sample size estimates for DSEG *θ* are considerably lower than those of MD NPH and WMH volume. For example, DSEG *θ* requires a sample size of 73 to detect a 30% change in SVD progression compared to 128 for MD NPH and 124 for WMH volume. This means that 41% fewer patients are required within a clinical trial for which a 30% change is expected when using the DSEG *θ* marker compared to WMH volume and 42% fewer compared to MD NPH.

**Table 7:** The predicted minimum sample size for a hypothetical trail of three-year duration assuming a balanced design with DSEG measurements taken annually to test a hypothetical treatment effect of 30, 25, 20 and 15% on the rate of *DSEG θ* change. For comparison, MD NPH and WMH volume values are also shown, taken from Benjamin et al. (2015) and Zeestraten et al. (2016).

	Hypothetical treatment effects			
	30%	25%	20%	15%
DSEG *θ* sample size	73	104	163	290
MD NPH (Zeestraten et al., 2016)	128	185	325	578
WMH volume (Benjamin et al., 2015)	124	178	279	496

## 4 Discussion

In this study we demonstrate that a novel DTI segmentation, DSEG, is a sensitive marker to assess the degree of brain damage in SVD. Although DSEG has been previously applied to the study of brain tumours, we demonstrate that it is also sensitive to subtle and gradual brain changes observed in SVD. DSEG spectra demonstrate sensitivity to individual differences in baseline measures as well as detecting differences in the rates of change over a three-year period. Segments most associated with tissue characteristics of GM and WM are shown to deviate from those characteristics of a healthy reference brain in SVD, even when the patient then remains stable over the subsequent three-year follow-up. For a patient with SVD who declines substantially over the follow-up period, the DSEG spectra demonstrate proportional changes in most segments, with a decrease in healthy GM and WM and an increase in WMH associated damage and CSF filled space. As such, DSEG can detect brain tissue changes characteristic of SVD.

As well as providing information about tissue diffusions characteristics in spectra, DSEG also provides a summary metric (DSEG *θ*) that characterises microstructural information derived from the whole brain. As with DSEG *θ*, other standard MRI measures including those from DTI histograms were sensitive to detect brain changes over the three-year follow-up. In contrast, cognitive function measured by EF and IPS did not demonstrate a significant decline in this analysis. This is in keeping with a previous report of no cognitive decline in patients with lacunar stroke followed for an average of three-years (Pearce et al., 2014). This may reflect the fact that MRI measures are more sensitive to decline in SVD patients when compared to cognitive measures. Alternatively, after the initial brain pathology that results in ‘caseness’ regarding the lacunar infarct condition and accompanying cognitive deficit, there may be a period in which there are further white matter brain changes, but cognition remains relatively stable. Additionally, the lack of longitudinal cognitive decline may also reflect in part learning effects when cognitive measures are repeatedly administered in longitudinal studies (Dikmen et al., 1999, Ferrer et al., 2005, Ferrer et al., 2004, Wilson et al., 2006).

One difference between the present study and previous investigations presenting derivations and investigation of total SVD burden scores is that we present a single measure derived from a single imaging modality rather than measures obtained from different MRI markers of SVD. For example, (Huijts et al., 2013, Klarenbeek et al., 2013, and Staals et al., 2014, Staals et al., 2015 previously used information from lacunar infarcts, WMH, CMB and enlarged perivascular spaces to obtain SVD burden scores. Our SVD burden score, DSEG *θ*, was related to all four conventional MRI markers of SVD used in this study (WMH load, TCV – representing cerebral atrophy, new lacunes and CMB), with an increase in DSEG *θ* being associated with higher SVD burden as measured by these markers. Furthermore, our SVD burden score was related to WM tissue microstructural histogram measures. An additional benefit of the DSEG technique, as also for the technique of Staals et al. (2015) which generated a latent variable from the full range of SVD markers, is that we also construct a summary variable that is not derived from dichotomised scores for SVD burden.

In this study linear mixed effects models were used as they allow for powerful exploration of the relationships between several hierarchical variables with multiple measures. LME allow for random variation at the individual level while fixed effects of time are taken in to account. This is in contrast to our previous paper using the SCANS cognitive data (Lawrence et al., 2015) in which three-year change was explored using linear regression analysis in which significant decline in EF was found. The rigorous statistical tests employed in the current study did not find significant decline in EF but this is likely due to the more conservative estimates of change imposed by LME analysis. However LME did allow full exploration of the relationships between change in brain markers and individual differences in gradients of cognitive function change despite the observation of no significant decline over the testing period. Despite the lack of a significant decline in cognitive function, univariable analysis demonstrated strong associations between change in MRI markers and change in cognition. For both EF and IPS, change in DSEG *θ*, standard DTI histogram metrics, and lesion measures (WMH load, lacunes, CMB), were associated with change in cognition. These results are in agreement with previous reports of change, with changes in DTI metrics, atrophy and incident lacunes being related to longitudinal decline in cognitive scores that measure attention, processing speed, EF and episodic memory (Jokinen et al., 2011, Jokinen et al., 2012, van Uden et al., 2015, van Norden et al., 2012). However, multivariable analyses demonstrate that DSEG *θ* remained a significant predictor of change in EF and IPS while standard MRI measures provided no further information, with the exception of new lacunes. Premorbid IQ contributed significantly to models for both EF and IPS suggesting a strong link between cognitive reserve (Stern, 2002) and subsequent impact of SVD on cognitive change. As premorbid IQ was positively related to cognition, this suggests that the more cognitive reserve present before onset of disease, the less the disease will affect cognition i.e. cognitive reserve serves as a protecting factor against cognitive decline.

In the LME models presented here, DSEG *θ* provides a very stable measure of microstructural brain change, as is shown by the highly significant goodness of fit (as assessed by the Wald test). As a result, when DSEG *θ* is considered in conjunction with other less stable MR markers in multivariable analysis, it provides the strongest predictor of cognitive change, in part because it has less noise in the model than other MR variables. In post-hoc sample size analysis, DSEG *θ* provided low sample size estimates ranging from 73 patients to detect a 30% treatment effect and 290 patients to detect a 15% treatment effect. This was compared to the sample size estimates for WMH volume and MD NPH, reported previously for the same SCANS patient sample over the same testing period (3-years). The results for these metrics were compared to DSEG *θ* because they provided the optimum sample size metrics for conventional MRI markers and DTI histogram markers of SVD progression (Benjamin et al., 2015 & Zeestraten et al., 2016). Compared to these, DSEG *θ* could detect the same change with 41–42% fewer patients in a clinical trial. These results suggest DSEG *θ* can be used as a clinical tool with a high degree of confidence that it will be sensitive to changes in SVD related brain structure.

The stability of DSEG *θ* is likely the result of all analysis being carried out in DTI native space across the entire cerebrum whereas DTI histogram measures rely on a hard segmentation of WM tissue that is dependent on a co-registration with a structural T1-weighted image and accurate automatic segmentation of GM and CSF boundaries, this will introduce noise in the data by including partial volume voxels at the border of the WM tissue mask images. DSEG is also an automatic segmentation technique and thus avoids any noise associated with manual segmentation and identification errors for WMH, lacunes and CMB. These results are similar to a recent report of a novel marker of SVD using the peak width of the skeletonised mean diffusivity (PSMD: Baykara et al., 2016) in which the DTI-derived marker demonstrated the strongest relationship with IPS in comparison to WMH volume, lacune volume and brain volume. However, DSEG may have the benefit of using information from the whole cerebrum (rather than WM only) and of incorporating both isotropic and anisotropic diffusion information rather than just MD. Furthermore, DSEG does not require any transformations of DTI data onto a skeletonised mean MD map, avoiding the introduction of noise through co-registration errors.

A limitation of the current analysis is that spatial information describing the location of SVD pathology has not been taken into account. It has already been highlighted by Staals et al. (2015) that SVD burden scores tend to neglect location information related to lacunes and CMB which have been shown to be important when exploring relationships between SVD markers and cognitive decline (Benisty et al., 2009, Poels et al., 2012). Future application of DSEG should address this by examining DSEG *θ* metrics by brain region or by combining DSEG with voxel based analysis. However, the current study demonstrates that DSEG provides a measure of SVD severity across the whole cerebrum, using a single imaging modality, without the need for co-registration of images across time points or into standard space, which would be necessary for more complex spatial analysis.

A further limitation is that the longitudinal cohort examined in this study is younger and less cognitively impaired compared to those patients who dropped out of the study after baseline testing. This suggests that there may be a selective attrition effect (Salthouse, 2009) in which patients who are least effected by the disease are more likely to remain in the study. This has implications for the generalizability of these results to all patients of SVD and suggests that the findings presented here are most applicable to those in the earlier or less severe stages of the disease. However, as DSEG *θ* has been shown to be sensitive to SVD progression in these “less affected” individuals, it is likely to be a useful tool in more severe cases of SVD as well.

As DSEG is related to all conventional MRI markers of SVD considered in the present study and is significantly associated with EF and IPS in predictive models, it may be used as a surrogate marker of SVD severity. By reducing whole cerebrum information to a single measure, DSEG *θ* can be used as a tool that avoids statistical power limitations due to multiple comparisons on a voxel or regional basis. DSEG measures have previously been shown to have good between site reproducibility (Jones et al., 2014), making DSEG *θ* a promising tool for large multi-site studies of SVD. Furthermore, sample size estimates suggest that DSEG *θ* has potential as a clinical tool, capable of detecting the effects of potential interventions with a high degree of confidence. Taken together, the promising performance of DSEG suggests that it could be a powerful tool for clinical trials, monitoring, and predicting disease progression in SVD patients as it shows strong relationships with the cognitive domains affected most by the disease.

The following is the supplementary data related to this article.

Supplementary Table 1 Median *p* and *q* values for each DSEG segment. Supplementary Table 1
