# Supplementary material for: Diffusion tensor image segmentation of the cerebrum provides a single measure of cerebral small vessel disease severity related to cognitive change
Source: Neuroimage Clin. 2017 Aug 15;16:330–42. doi: 10.1016/j.nicl.2017.08.016 (PMC5568143; doi:10.1016/j.nicl.2017.08.016)
Supplement: Supplementary Table 1 — Median p and q values for each DSEG segment. [file mmc1.docx]

**Supplementary Table 1:** Median *p* and *q* values for each DSEG segment.

| *Segment* | *p* (Χ 10^-3^ mm^2^ s^-1^) | *q (*Χ 10^-3^ mm^2^ s^-1^) |
| --- | --- | --- |
| 1 | 0.44×10^-3^ | 0.13×10^-4^ |
| 2 | 0.57×10^-3^ | 0.26×10^-4^ |
| 3 | 0.62×10^-3^ | 0.36×10^-4^ |
| 4 | 0.64×10^-3^ | 0.49×10^-4^ |
| 5 | 0.70×10^-3^ | 0.75×10^-4^ |
| 6 | 0.77×10^-3^ | 0.17×10^-4^ |
| 7 | 1.38×10^-3^ | 0.24×10^-4^ |
| 8 | 2.53×10^-3^ | 0.43×10^-4^ |
| 9 | 0.70×10^-3^ | 0.07×10^-5^ |
| 10 | 1.13×10^-3^ | 0.08×10^-5^ |
| 11 | 1.59×10^-3^ | 0.07×10^-5^ |
| 12 | 2.10×10^-3^ | 0.08×10^-5^ |
| 13 | 2.66×10^-3^ | 0.09×10^-5^ |
| 14 | 3.32×10^-3^ | 0.13×10^-4^ |
| 15 | 3.97×10^-3^ | 0.31×10^-4^ |
| 16 | 4.30×10^-3^ | 0.15×10^-4^ |
